# Supplementary figures and images for: From tiny to immense: Geological spotlight on the Alexander Mosaic (National Archaeological Museum of Naples, Italy) using non-invasive in situ analyses
Source: PLoS One. 2025 Jan 15;20(1):e0315188. doi: 10.1371/journal.pone.0315188 (PMC11734927; doi:10.1371/journal.pone.0315188)

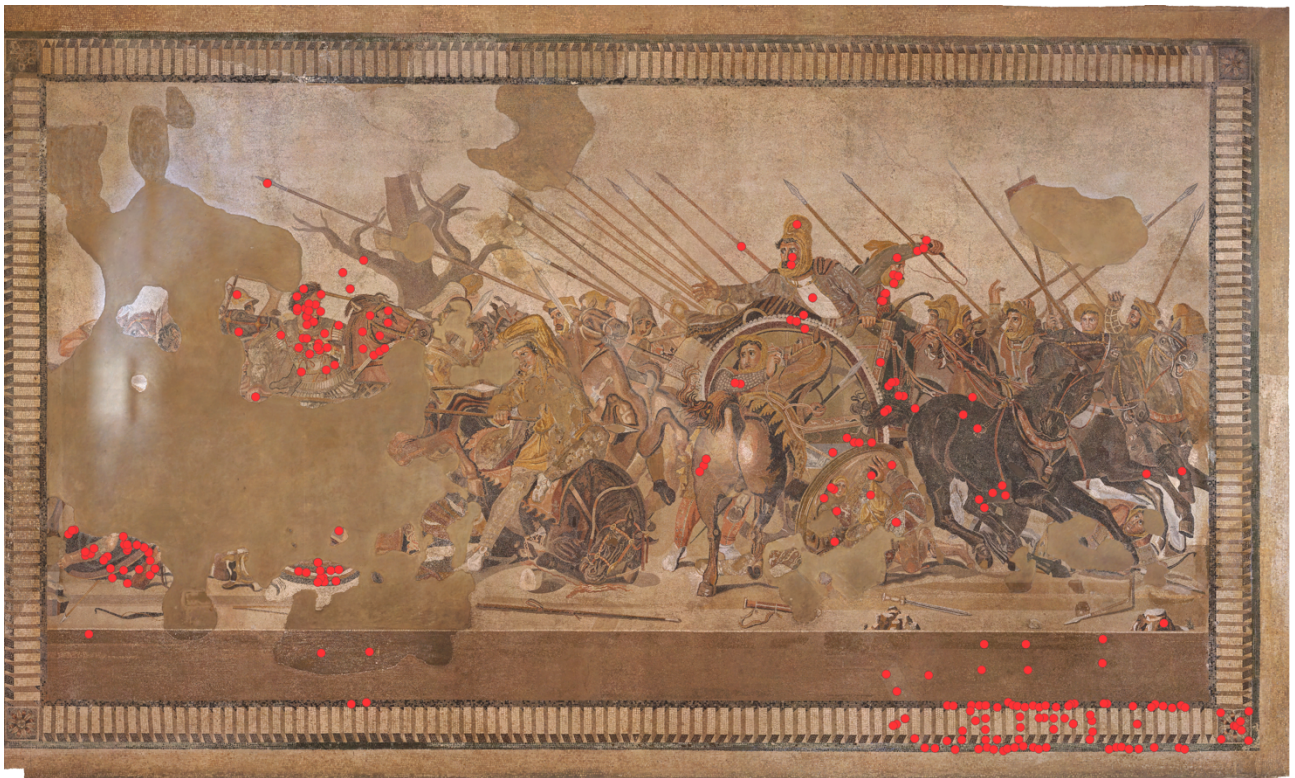

**S1 Fig**

Supplement: S1 Fig — (PDF) [file pone.0315188.s001.pdf]

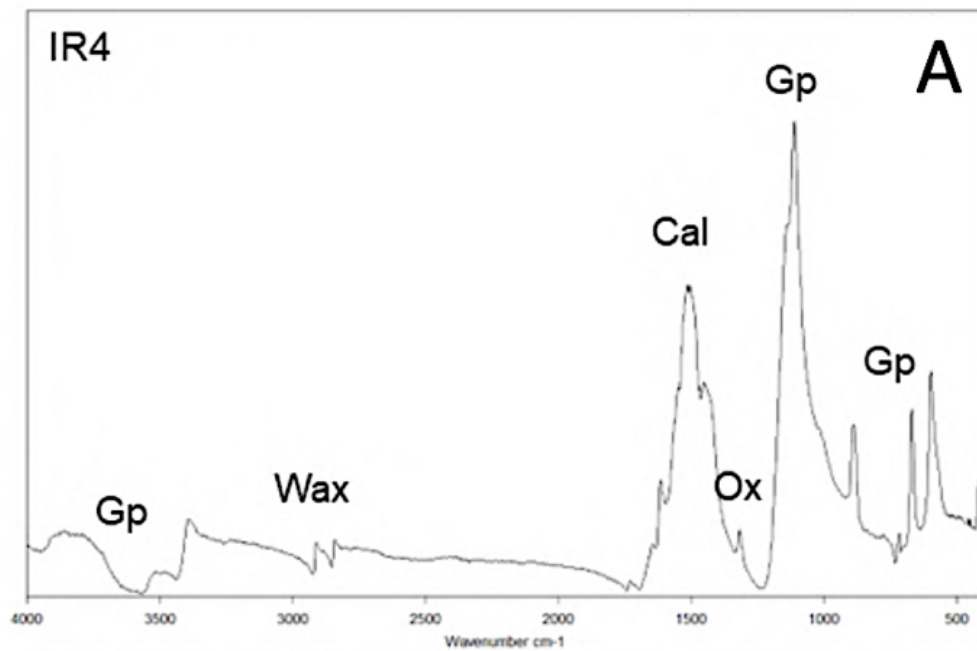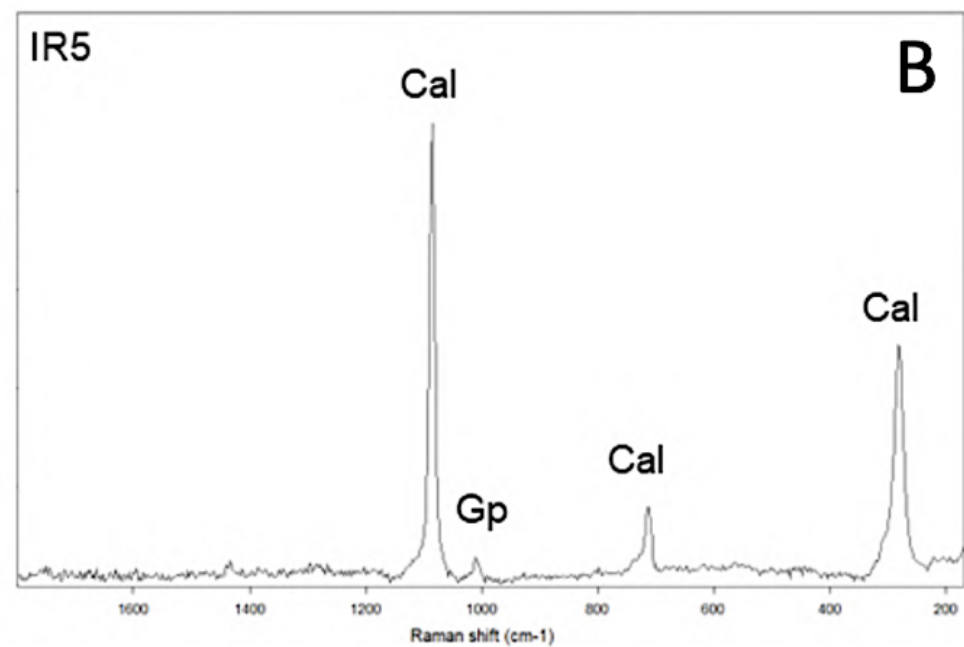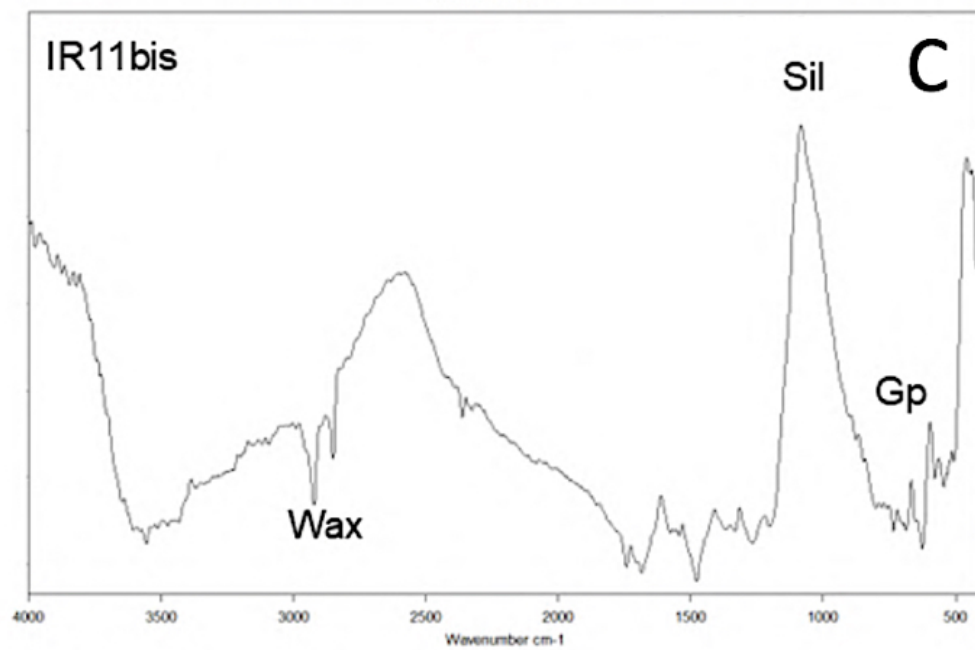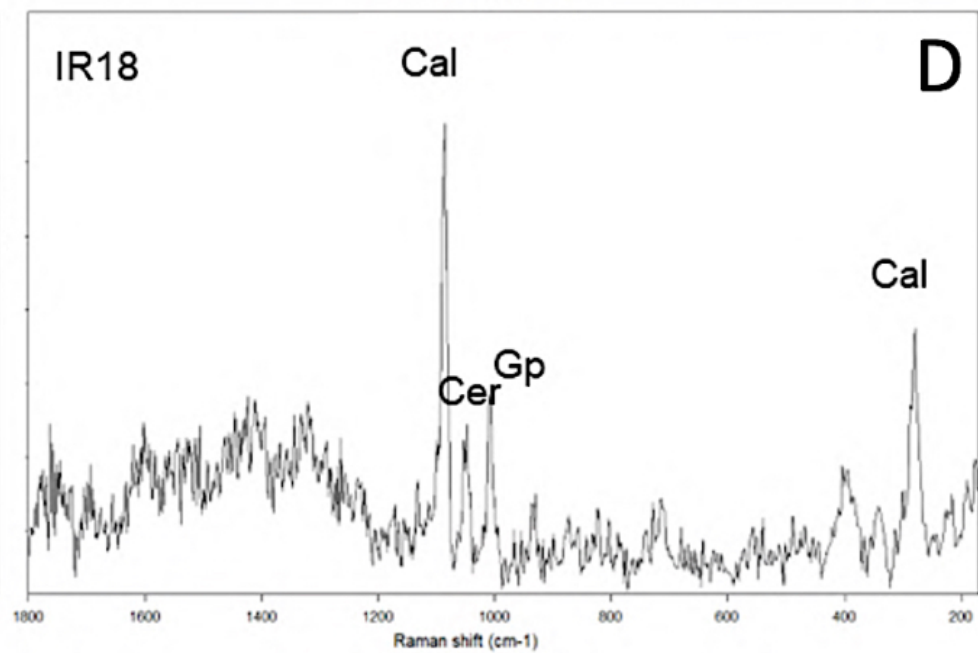

**S2 Fig**

Supplement: S2 Fig — (PDF) [file pone.0315188.s002.pdf]

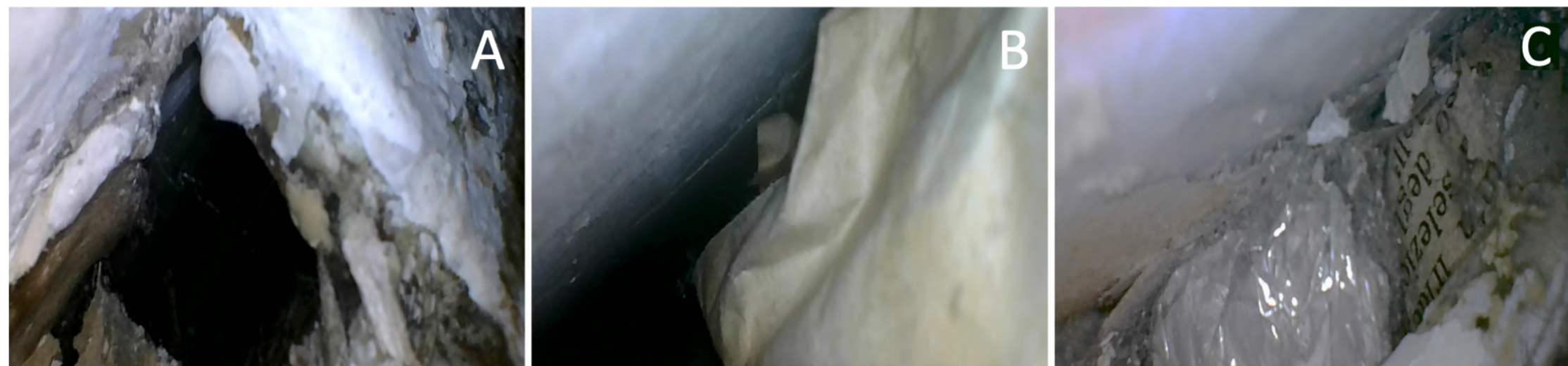

S3 Fig

Supplement: S3 Fig — Evident spaces filled with binder depositions (A), probably composed of vinyl or gypsum-based substances, along with paper, including newspaper (B, C, D). (PDF) [file pone.0315188.s003.pdf]

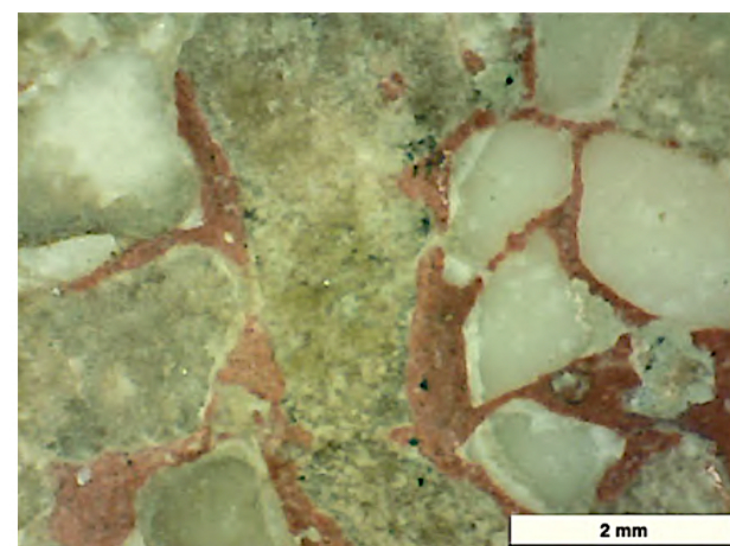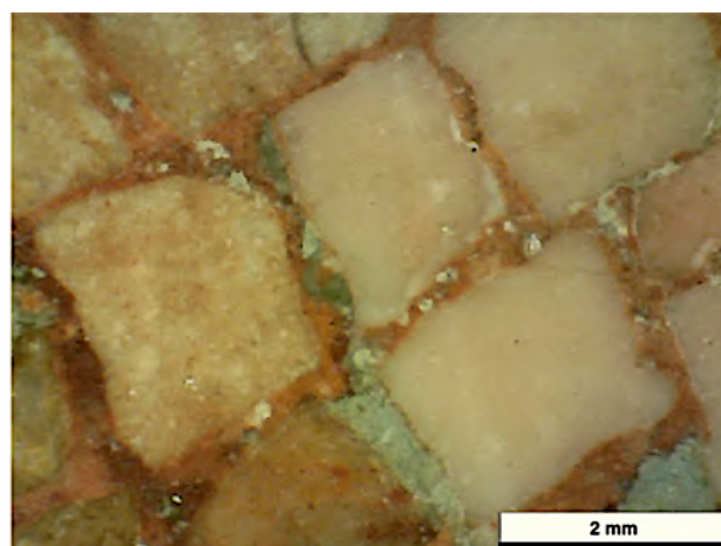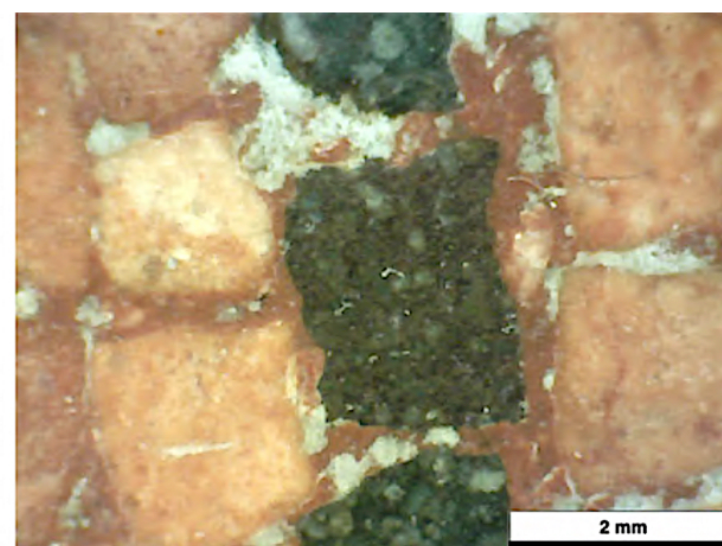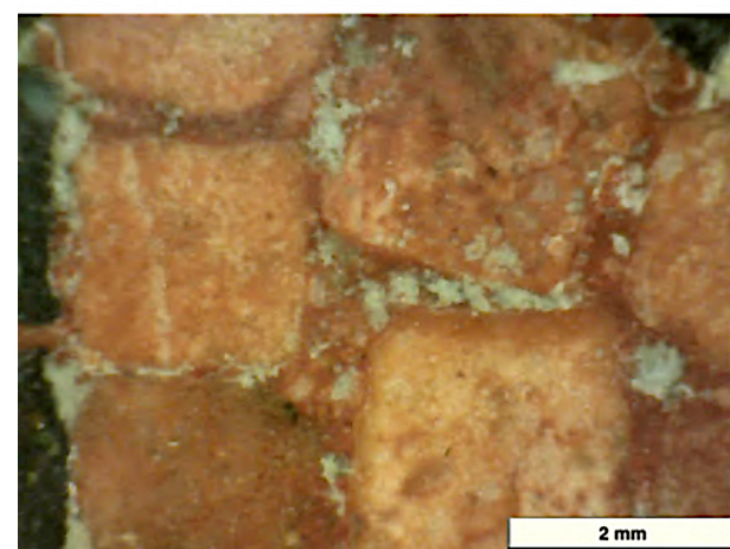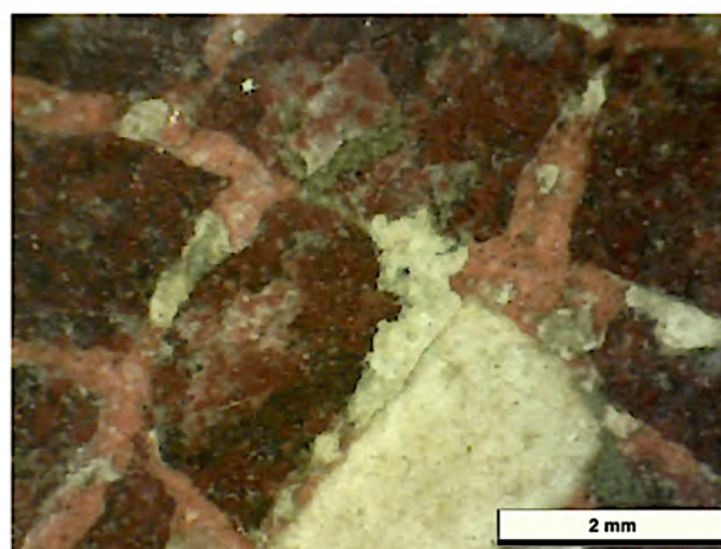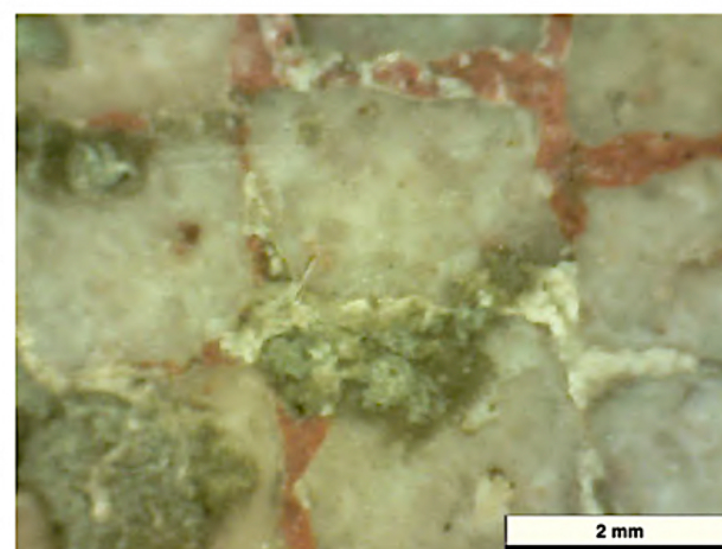

S4 Fig

Supplement: S4 Fig — (PDF) [file pone.0315188.s004.pdf]
